# Enhancing Postprandial Hyperglycemia Detection Sensitivity in Individuals With Impaired Glucose Tolerance by Incorporating 1,5-Anhydroglucitol Into Diagnostic Testing: A Multicenter and Prospective Observational Study

**DOI:** 10.7759/cureus.83297

**Published:** 2025-05-01

**Authors:** Kensaku Fukunaga, Toshihiro Kobayashi, Hitomi Imachi, Shin Horikawa, Yasuyoshi Iwata, Takanobu Saheki, Takafumi Yoshimura, Seisuke Sato, Rathana Ly, Koji Murao

**Affiliations:** 1 Department of Endocrinology and Metabolism, Kagawa University, Kita-gun, JPN; 2 Internal Medicine, Kinashi Obayashi Hospital, Takamatsu, JPN; 3 Internal Medicine, Kagawa Rosai Hospital, Marugame, JPN

**Keywords:** 1, 5-anhydroglucitol, impaired glucose tolerance, postprandial glucose spikes, type 2 diabetes

## Abstract

Objective: Prospective multicenter trials evaluating the sensitivity of 1,5-anhydroglucitol (1,5-AG) for detecting postprandial hyperglycemia in individuals with impaired glucose tolerance during health checkups in regional Japanese cities are limited. This study aims to address this gap through a comprehensive investigation.

Methods: Sixty-two participants with glycated hemoglobin (HbA1c) levels of 5.6-6.5% underwent a 75 g oral glucose tolerance test. Our primary objective was to evaluate the sensitivity of 1,5-AG compared to HbA1c in detecting ≥180 mg/dL glucose levels after an hour. Secondary endpoints are the sensitivity and specificity of the detection of load hyperglycemia.

Results: The area-under-the-curve analysis indicated that 1,5-AG was not more sensitive than HbA1c in detecting ≥180 mg/dL glucose levels (0.543 vs. 0.686, p = 0.236). However, combining 1,5-AG with fasting blood glucose (FBG: 27.8% vs. FBG + 1,5-AG: 72.2%, p = 0.004) or HbA1c (HbA1c: 44.4% vs. HbA1c + 1,5-AG: 72.2%, p = 0.042) improved the sensitivity of detecting glucose spikes, suggesting that 1,5-AG may be a valuable addition to standard screening protocols for reducing undetected postprandial hyperglycemia, a risk factor for diabetes and cardiovascular diseases.

Conclusion: These findings support the potential of 1,5-AG as an early marker of postprandial hyperglycemia during routine health checks.

## Introduction

“Glucose spikes” are rapid increases in blood glucose levels after meals, which are significant contributors to diabetes and atherosclerosis, and typically arise from impaired glucose tolerance (IGT). Postprandial hyperglycemia and elevated blood glucose levels after eating are significant risk factors for cardiovascular diseases. The Hisayama study [1] conducted in Japan has shown that individuals with IGT, defined as two-hour blood glucose levels of 140-199 mg/dL during a 75 g oral glucose tolerance test (OGTT), have an increased risk of coronary artery disease than those with normal glucose tolerance. Similarly, the Funagata study [2], which followed individuals aged 35 years and older, found that those with IGT or diabetes were more likely to experience cardiovascular events than those with normal glucose levels. Notably, within the borderline glucose tolerance group, individuals with IGT had a higher incidence of cardiovascular events than those with impaired fasting glucose. The Honolulu Heart Study [3] also supported these findings, showing an increased risk of coronary artery disease associated with higher blood glucose levels one hour after the glucose load. Therefore, monitoring postprandial glucose levels is critical. However, because glucose spikes occur shortly after eating, they are often missed in fasting blood glucose (FBG) or glycated hemoglobin (HbA1c) measurements, which are commonly used in routine health check-ups.

These short-lived spikes are often missed in standard health evaluations, which usually only measure FBG and HbA1c levels [4]. Traditionally, in individuals without diabetes, blood glucose levels peak around 60 minutes after eating, rarely exceeding 140 mg/dL, and usually return to pre-meal levels within two to three hours [5,6]. However, recent developments in continuous glucose monitoring (CGM) technology have altered this understanding. Another study comprising 57 adults (median age, 51 years) without a diabetes history using CGM technology found that even those with normal glucose levels or early signs of diabetes exhibited significant daily fluctuations in blood glucose levels. This variability indicates a high risk of developing diabetes [7]. A previous study involving 36 overweight or obese men without diabetes [8] revealed that those who exercised less and snacked frequently had an increased risk of attaining glucose levels of 200 mg/dL or higher. This suggests that high postprandial blood glucose levels, which are often undetected in non-diabetic individuals, may be more common than previously believed.

1,5-Anhydroglucitol (1,5-AG) is a useful biomarker for assessing hyperglycemia (>160-180 mg/dL) over the past one to two weeks using random blood samples. Previous research has indicated that 1,5-AG can detect glucose spikes more accurately than HbA1c in diabetic patients with relatively good blood glucose control, as monitored using continuous glucose monitoring devices [9]. In addition, a long-term study of 2,095 residents in Japan (991 men and 1,104 women, mean age 58.5 years) showed that low levels of 1,5-AG in individuals without diabetes, particularly men, were significantly associated with the risk of developing cardiovascular disease [10]. This suggests that 1,5-AG is sensitive in detecting high postprandial blood glucose levels and predicting related complications.

Despite these insights, limited studies are available comparing the diagnostic accuracy of 1,5-AG with HbA1c in individuals with IGT, particularly those with HbA1c levels between 5.6% and 6.5% identified during routine health screening. In this study, we conducted an OGTT on individuals with HbA1c levels in this range who were flagged for IGT during voluntary health check-ups. Since routine health check-ups often overlook postprandial blood glucose spikes, we aimed to evaluate the sensitivity of 1,5-AG in detecting glucose spikes in these individuals.

## Materials and methods

Study design and protocols

**Figure 1:**
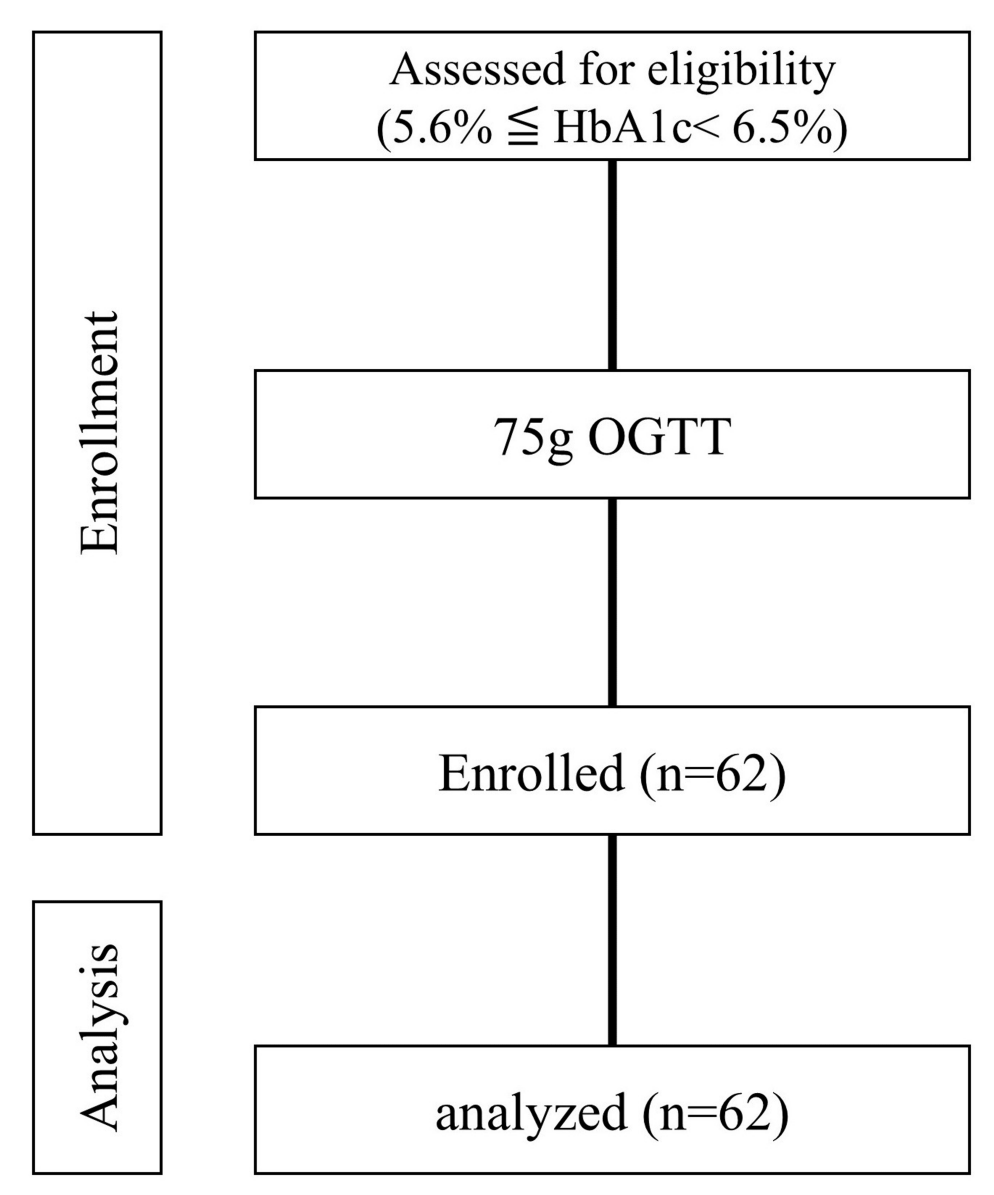
Flowchart representing the patient selection process.

This was a multicenter and prospective observational study involving 62 participants who were identified as having IGT (HbA1c levels: 5.6-6.4%) and underwent a 75 g OGTT during voluntary health check-ups at Kagawa University Hospital, Kinashi Obayashi Hospital, Kagawa Rosai Hospital, and Takamatsu Red Cross Hospital, between 2021 and 2023. All participants provided consent for the measurement of 1,5-AG levels (Figure 1). To minimize potential sources of bias, participants were required to meet strict inclusion criteria (HbA1c levels: 5.6-6.5%), and standardized testing protocols (75 g OGTT) were applied uniformly. The primary objective was to test whether the area under the receiver operating characteristic (ROC) curve (AUC) for 1,5-AG in detecting a one-hour OGTT glucose level of 180 mg/dL exceeded 0.5. Secondary objectives included evaluating the sensitivity and specificity of 1,5-AG, HbA1c, FBG, and their combinations to detect increases in post-load blood glucose levels: (i) a one-hour OGTT glucose levels of 180 mg/dL or two-hour glucose levels of 140 mg/dL, and (ii) a one-hour OGTT glucose levels of 155 mg/dL or two-hour glucose levels of 140 mg/dL. The rationale for our choice of these criteria is that a one-hour OGTT cut-off of 180 mg/dL is a key marker for the early detection of atherosclerosis and diabetes risk [5]. The two-hour OGTT value of 140 mg/dL was established by the World Health Organization in 1980 and is widely used to diagnose IGT. The Funagata Diabetes Study [2] showed that IGT contributes to the onset of diabetes and atherosclerosis. Similarly, the Hisayama Study [1] reported that individuals with IGT, defined as a two-hour OGTT glucose level of 140 mg/dL or higher, have an increased risk of coronary artery disease compared with those with normal glucose tolerance (NGT). A one-hour OGTT glucose level of 155 mg/dL is also an effective indicator of cardiovascular risk. Several reports have demonstrated that individuals attaining glucose levels of 155 mg/dL or higher one-hour after glucose loading have an increased risk of developing diabetes and cardiovascular diseases, supporting its use in risk evaluation [11,12]. Blood samples were collected from all participants after a 10-hour overnight fast.

Definition and diagnostic criteria

In this study, a cut-off value of 21 μg/mL for 1,5-AG was determined based on the results of the ROC curve analysis. As part of our diagnostic criteria, this value was used to identify postprandial hyperglycemia in individuals with IGT. The cut-off values for HbA1c and FBG were set according to the guidelines for health check-ups in Japan, where HbA1c ≥ 6.0% and FBG ≥ 110 mg/dL were considered thresholds for further examination. These values were chosen based on their use in routine health screening as indicators of potential retesting.

Ethical approval

This study adhered to the Declaration of Helsinki (latest version) and Ethical Guidelines for Medical and Health Research Involving Human Subjects. It was approved by the Kagawa University Medical Ethics Committee (approval number: 2020-163, recognition date: January 15, 2021). This study was also registered with the UMIN Clinical Trials Registry (trial registration number: UMIN000047552).

Informed consent

Informed consent was obtained from all individual participants included in the study.

Statistical analysis

Based on the primary objective of evaluating the AUC for 1,5-AG in detecting a one-hour OGTT glucose level ≥180 mg/dL, sample size calculations assumed a two-sided significance level of 5% and 90% statistical power. Depending on expected AUC values and population proportions reaching the 180 mg/dL threshold, the required sample size varied. With an expected 50% prevalence and an AUC of 0.75, 60 participants were deemed sufficient.

Participants with HbA1c >6.5% were excluded to avoid bias from severe glucose dysregulation. There were no missing data; thus, no imputation was needed. Statistical analyses were performed using IBM SPSS Statistics for Windows, Version 28.0.1 (released 2021, IBM Corp., Armonk, NY). Quantitative variables were summarized as counts (%), means ± SD, or medians (IQR). Student’s t-tests and McNemar’s test were used to compare numerical data and sensitivity/specificity, respectively. Diagnostic markers were analyzed using ROC curves to assess AUC, sensitivity, and specificity. Adjusted risk estimates were not calculated as the focus was diagnostic performance rather than outcomes. Subgroup analyses were not conducted due to the limited sample size.

## Results

Participant characteristics

Figure 1 shows a flowchart of the participant selection. The characteristics of the 62 participants included in the analysis are listed in Table 1. The average age of the participants was 50.2 ± 8.0 years, with 29% being female and 71% male. The mean weight was 66.1 ± 13.8 kg, and the average BMI was 23.4 ± 3.7. The FBG level averaged 98.8 ± 8.8 mg/dL, the OGTT one-hour glucose level was 142.2 ± 44.8 mg/dL, and the OGTT two-hour glucose level was 119.8 ± 33.4 mg/dL. The Homeostatic Model Assessment for Insulin Resistance (HOMA-R) value was 1.86 ± 1.32, HbA1c was 5.8 ± 0.2%, and serum 1,5-AG levels were 24.0 ± 7.9 μg/mL. The other metrics are listed in Table 1.

**Table 1:** Characteristics of the 62 participants.

Characteristic	Mean (SD)	Median (Range)
Age (years old)	50.2 (8.0)	50.0 (35.0, 71.0)
Gender	Female 18 (29%)	Male 44 (71%)
Height (cm)	167.6 (9.2)	168.8 (143.6, 184.0)
Body weight (kg)	66.1 (13.8)	65.0 (39.9, 99.9)
Waist circumference (cm)	83.1 (10.2)	81.0 (66.2, 110.0)
BMI (kg/m2)	23.4 (3.7)	22.9 (18.2, 35.9)
Grip strength (R)	38.6 (11.2)	40.0 (15.0, 58.0)
Grip strength (L)	37.2 (10.8)	39.0 (13.0, 55.0)
Systolic blood pressure (mmHg)	120.5 (12.5)	122.0 (93.0, 147.0)
Diastolic blood pressure (mmHg)	76.3 (11.5)	78.0 (44.0, 103.0)
Fe	99.1 (40.5)	101.0 (28.0, 195.0)
T-cho (mg/dL)	222.1 (33.3)	218.0 (162.0, 310.0)
TG (mg/dL)	111.4 (58.8)	103.0 (29.0, 373.0)
HDL-C (mg/dL)	69.7 (18.9)	66.0 (43.0, 117.0)
LDL-C (mg/dL)	135.5 (28.0)	133.0 (42.0, 200.0)
nonHDL-C (mg/dL)	152.5 (31.6)	150.0 (47.0, 224.0)
AST (U/L)	22.7 (5.4)	21.5 (14.0, 45.0)
ALT (U/L)	21.4 (13.5)	17.5 (7.0, 77.0)
γ-GTP (IU)	29.2 (23.4)	22.0 (9.0, 155.0)
Fasting blood glucose (mg/dL)	98.8 (8.8)	99.0 (80.0, 119.0)
Postprandial BG (1hour) (mg/dL)	142.2 (44.8)	146.5 (48.0, 222.0)
Postprandial BG (2hour) (mg/dL)	119.8 (33.4)	115.0 (58.0, 231.0)
Fasting IRI (μU/mL)	7.4 (4.9)	6.3 (1.2, 23.8)
1,5-AG (μg/mL)	24.0 (7.9)	23.8 (8.4, 39.7)
HbA1c (NGSP) (%)	5.8 (0.2)	5.7 (5.6, 6.4)
HOMA-IR	1.86 (1.32)	1.53 (0.28, 6.46)
Cre (mg/dL)	0.87 (0.17)	0.88 (0.56, 1.30)
eGFR (mL/min/1.73m2)	70.2 (12.1)	69.0 (42.0, 108.0)
Urinary acid (mg/dL)	5.4 (1.4)	5.6 (2.8, 8.3)

Comparison of areas under ROCs

**Figure 2:**
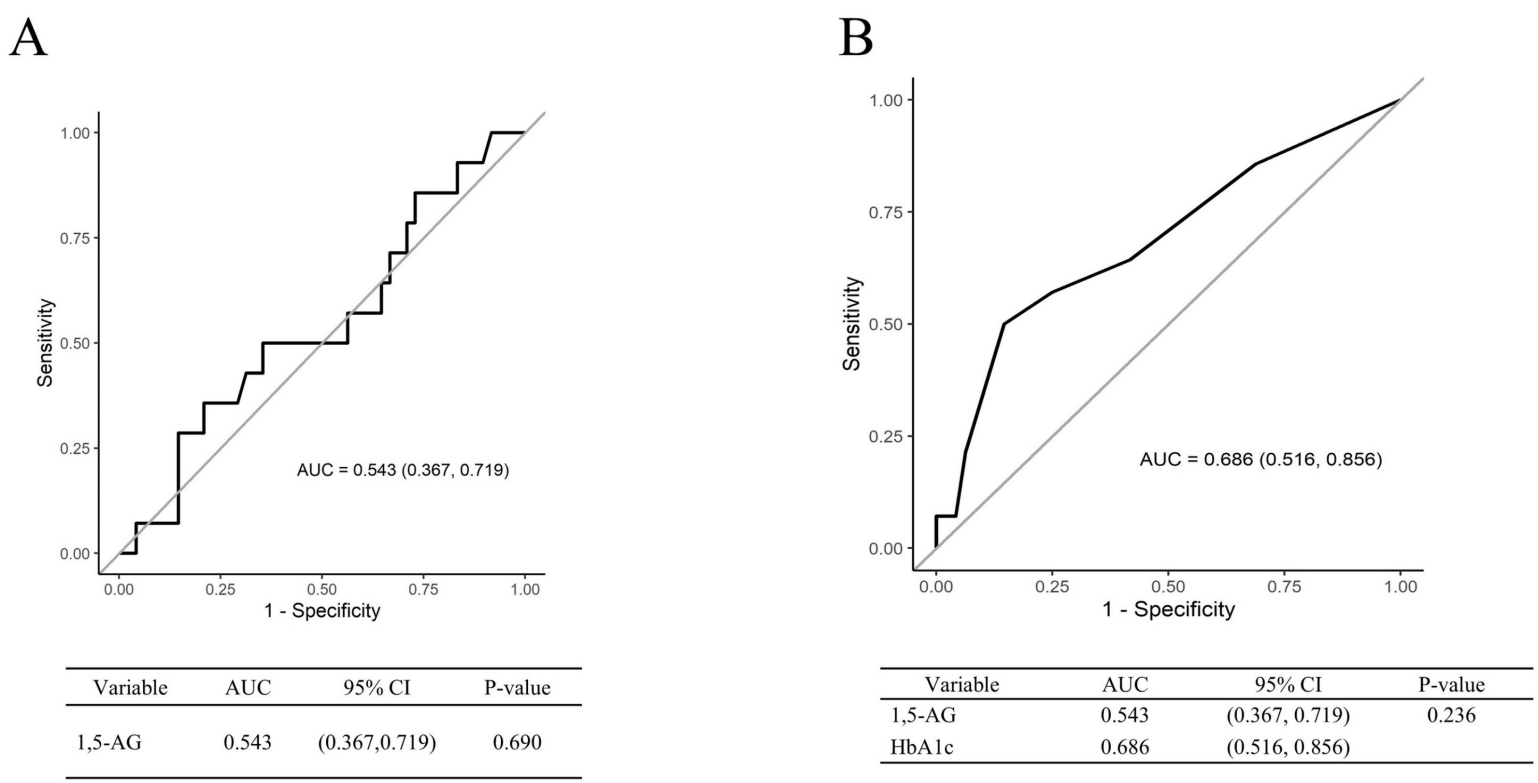
ROC curves: A) for 1,5-AG in detecting OGTT one-hour blood glucose levels of 180 mg/dL, B) for HbA1c in detecting OGTT one-hour blood glucose levels of 180 mg/dL.

The primary outcome analysis focused on testing whether the AUC for 1,5-AG in detecting an OGTT one-hour glucose level of 180 mg/dL exceeded 0.5. The average 1,5-AG levels in the participants was 24.0 ± 7.9 μg/mL. The results indicated that the AUC for 1,5-AG was 0.543 (95% confidence interval: 0.367-0.719), and the p-value for the hypothesis test was 0.690, showing no statistically significant difference in the primary analysis (Figure 2A).

For secondary outcomes, we examined the performance of each indicator. The AUC for HbA1c in detecting the OGTT one-hour glucose level of 180 mg/dL was 0.686. Comparison of the AUCs between 1,5-AG and HbA1c yielded a p-value of 0.236, indicating no significant difference in their ability to detect postprandial peaks (Figure 2B). Similarly, for detecting an OGTT two-hour glucose level of 140 mg/dL, 1,5-AG had an AUC of 0.644, whereas HbA1c had an AUC of 0.656, with no significant differences (Figure 2C, 2D).

**Figure 3:**
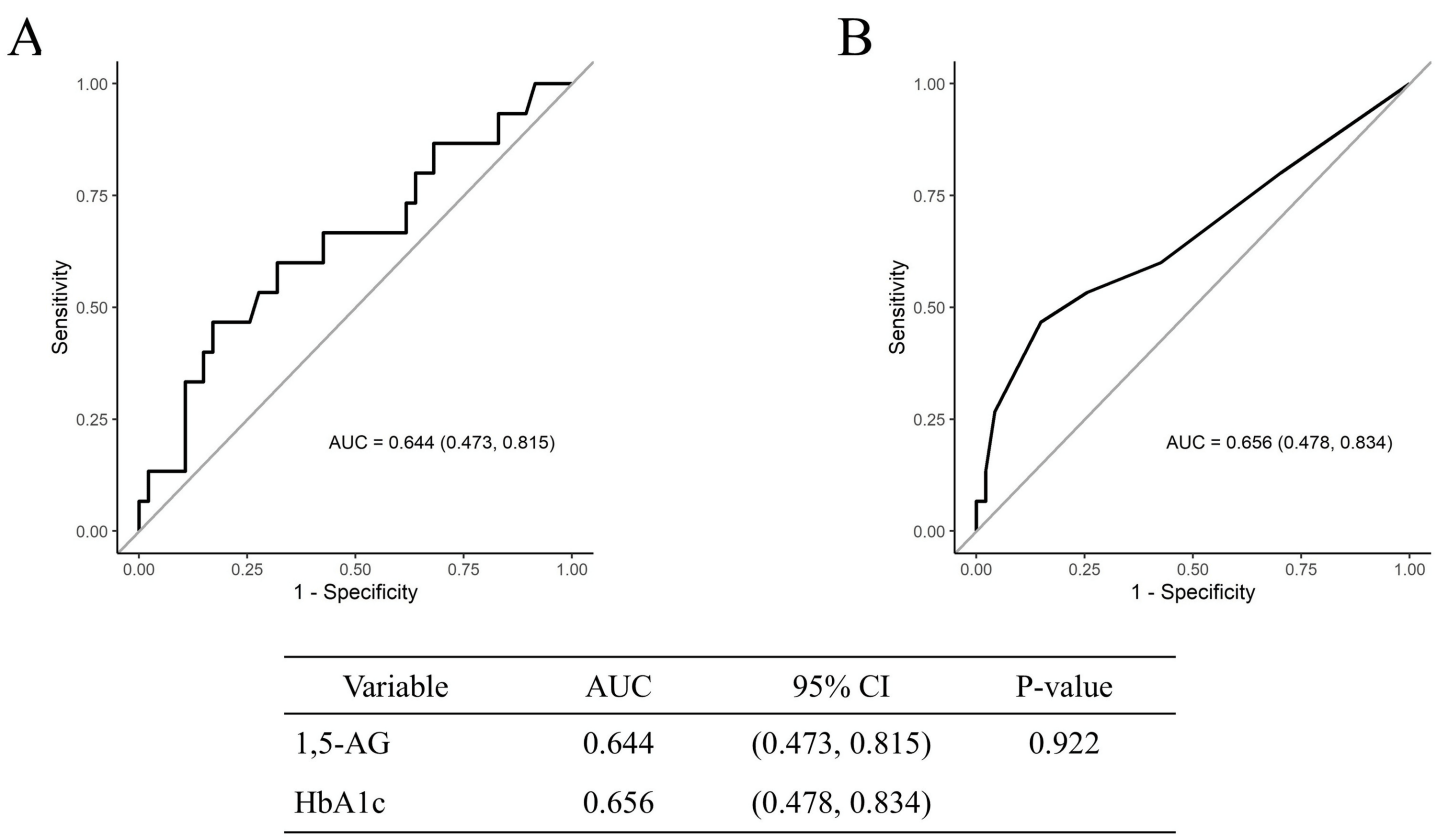
ROC curves A) for 1,5-AG in detecting OGTT two-hour blood glucose levels of 140 mg/dL, B) for HbA1c in detecting OGTT two-hour blood glucose levels of 140 mg/dL.

Sensitivity and specificity for detecting post-OGTT blood glucose increases

We evaluated the sensitivity and specificity of each indicator to compare their diagnostic capabilities. As mentioned above, the cut-off values used to detect 1,5-AG, HbA1c, and FBG were ≤21 μg/mL, ≥6.0%, and ≥110 mg/dL, respectively. We initially compared the diagnostic ability for the OGTT one-hour glucose levels of ≥180 mg/dL or two-hour glucose levels of ≥140 mg/dL among the three groups. Although 1,5-AG showed a tendency toward higher sensitivity, there were no significant differences among the groups in these individual comparisons (Table 2). In terms of specificity, FBG levels had the highest specificity, followed by HbA1c and then 1,5-AG levels (Table 3).

**Table 2:** Comparison of sensitivity for detecting OGTT one-hour blood glucose levels of 180 mg/dL or two-hour levels of 140 mg/dL among the three groups.

inspection item 1		inspection item 2		inspection item 3		Sensitivity 1	Sensitivity 2	Sensitivity 3	p-value
HbA1c	vs	1,5-AG	vs	FBG		44.4%	55.6%	27.8%	0.232
HbA1c	vs	HbA1c+1,5-AG	vs	HbA1c+FBG		44.4%	72.2%	55.6%	0.042
FBG	vs	FBG+1,5-AG	vs	FBG+HbA1c		27.8%	72.2%	55.6%	0.004

**Table 3:** Comparison of specificity for detecting OGTT one-hour blood glucose levels of 180 mg/dL or two-hour levels of 140 mg/dL among the three groups.

inspection item 1		inspection item 2		inspection item 3		Specificity 1	Specificity 2	Specificity 3	p-value
HbA1c	vs.	1,5-AG	vs.	FBG		86.4%	68.2%	93.2%	0.006
HbA1c	vs.	HbA1c+1,5-AG	vs.	HbA1c+FBG		86.4%	59.1%	84.1%	<0.01
FBG	vs.	FBG+1,5-AG	vs.	FBG+HbA1c		93.2%	61.4%	84.1%	<0.01

We examined the detection capabilities of individual indicators and their combinations across the two groups (Table 4). When using HbA1c as the base, combining it with 1,5-AG significantly increased the sensitivity compared to HbA1c alone, although the specificity was higher for HbA1c alone. When FBG levels were used as the base, combining it with 1,5-AG levels, the sensitivity improved compared with FBG levels alone, but the specificity remained higher with FBG levels alone. Evaluation of the effects of combining different pairs of markers revealed that combining FBG levels with other indicators significantly enhanced specificity.

**Table 4:** Comparison of sensitivity and specificity for detecting OGTT one-hour blood glucose levels of 180 mg/dL or two-hour levels of 140 mg/dL between the two groups.

inspection item 1		inspection item 2		Sensitivity 1	Sensitivity 2	p		Specificity 1	Specificity 2	p-value
HbA1c	vs	1,5-AG		44.4%	55.6%	1		86.4%	68.2%	1
HbA1c	vs	FBG		44.4%	27.8%	0.925		86.4%	93.2%	1
HbA1c	vs	HbA1c+1,5-AG		44.4%	72.2%	0.037		86.4%	59.1%	<0.001
HbA1c	vs	HbA1c＋FBG		44.4%	55.6%	0.952		86.4%	84.1%	1
FBG	vs	1,5-AG		27.8%	55.6%	0.268		93.2%	68.2%	0.268
FBG	vs	FBG+HbA1c		27.8%	55.6%	0.124		93.2%	84.1%	0.662
FBG	vs	FBG+1,5-AG		27.8%	72.2%	0.003		93.2%	61.4%	0.002
FBG	vs	HbA1c+1,5-AG		27.8%	72.2%	0.003		93.2%	59.1%	<0.001
1,5-AG	vs	1,5-AG+HbA1c		55.6%	72.2%	0.897		68.2%	59.1%	1
1,5-AG	vs	1,5-AG＋FBG		55.6%	72.2%	0.897		68.2%	61.4%	1
1,5-AG	vs	HbA1c＋FBG		55.6%	55.6%	1		68.2%	84.1%	0.118
HbA1c+1,5-AG	vs	HbA1c＋FBG		72.2%	55.6%	0.401		59.1%	84.1%	0.001
HbA1c+1,5-AG	vs	FBG+1,5-AG		72.2%	72.2%	1		59.1%	61.4%	1
HbA1c＋FBG	vs	FBG+1,5-AG		55.6%	72.2%	0.662		84.1%	61.4%	0.007

We also compared the indicators for detecting OGTT one-hour glucose levels of 155 mg/dL or two-hour glucose levels of 140 mg/dL, as shown in Tables 5-6. In comparison among the three groups, 1,5-AG levels tended to show higher sensitivity, whereas the specificity was the highest for FBG levels, followed by HbA1c and 1,5-AG levels (Tables 5-6). When examining the detection capabilities between the two groups, using HbA1c as the base and combining it with 1,5-AG levels significantly increased the sensitivity compared to HbA1c alone, while the specificity was higher for HbA1c alone (Table 7). Consistent with earlier trends, combining 1,5-AG levels improved sensitivity, whereas the group that combined FBG showed higher specificity.

**Table 5:** Comparison of sensitivity for detecting OGTT one-hour blood glucose levels of 155 mg/dL or two-hour levels of 140 mg/dL among the three groups.

inspection item 1		inspection item 2		inspection item 3		Sensitivity 1	Sensitivity 2	Sensitivity 3	p-value
HbA1c	vs.	1,5-AG	vs.	FBG		34.6%	50.0%	19.2%	0.049
HbA1c	vs.	HbA1c+1,5-AG	vs.	HbA1c+FBG		34.6%	61.5%	42.3%	0.008
FBG	vs.	FBG+1,5-AG	vs.	FBG+HbA1c		19.2%	61.5%	42.3%	0.044

**Table 6:** Comparison of specificity for detecting OGTT one-hour blood glucose levels of 155 mg/dL or two-hour levels of 140 mg/dL among the three groups.

inspection item 1		inspection item 2		inspection item 3		Specificity 1	Specificity 2	Specificity 3	p-value
HbA1c	vs	1,5-AG	vs	FBG		86.1%	69.4%	91.7%	0.039
HbA1c	vs	HbA1c+1,5-AG	vs	HbA1c+FBG		86.1%	58.3%	83.3%	<0.001
FBG	vs	FBG+1,5-AG	vs	FBG+HbA1c		91.7%	61.1%	83.3%	0.003

**Table 7:** Comparison of sensitivity and specificity for detecting OGTT one-hour blood glucose levels of 155 mg/dL or two-hour levels of 140 mg/dL between the two groups.

inspection item 1		inspection item 2		Sensitivity 1	Sensitivity 2	p		Specificity 1	Specificity 2	p-value
HbA1c	vs	1,5-AG		34.6%	50.0%	1		86.1%	69.4%	1
HbA1c	vs	FBG		34.6%	19.2%	0.925		86.1%	91.7%	1
HbA1c	vs	HbA1c+1,5-AG		34.6%	61.5%	0.037		86.1%	58.3%	<0.001
HbA1c	vs	HbA1c＋FBG		34.6%	42.3%	0.952		86.1%	83.3%	1
FBG	vs	1,5-AG		19.2%	50.0%	0.268		91.7%	69.4%	0.268
FBG	vs	FBG+HbA1c		19.2%	42.3%	0.124		91.7%	83.3%	0.662
FBG	vs	FBG+1,5-AG		19.2%	61.5%	0.008		91.7%	61.1%	0.008
FBG	vs	HbA1c+1,5-AG		19.2%	61.5%	0.003		91.7%	58.3%	<0.001
1,5-AG	vs	1,5-AG+HbA1c		50.0%	61.5%	1		69.4%	58.3%	0.944
1,5-AG	vs	1,5-AG＋FBG		50.0%	61.5%	1		69.4%	61.1%	1
1,5-AG	vs	HbA1c＋FBG		50.0%	42.3%	1		69.4%	83.3%	0.463
HbA1c+1,5-AG	vs	HbA1c＋FBG		61.5%	42.3%	0.401		58.3%	83.3%	0.001
HbA1c+1,5-AG	vs	FBG+1,5-AG		61.5%	61.5%	1		58.3%	61.1%	1
HbA1c＋FBG	vs	FBG+1,5-AG		42.3%	61.5%	0.662		83.3%	61.1%	0.007

## Discussion

In this study, we found that using 1,5-AG alongside HbA1c or FBG increased the sensitivity in detecting high blood glucose levels after an OGTT compared to using either measure alone. This was true for both one-hour (180 mg/dL) and two-hour (140 mg/dL) glucose levels. This combination reduces the chance of missing postprandial glucose spikes linked to the progression of diabetes and cardiovascular diseases. This suggests that 1,5-AG may serve as an effective early marker for the detection of postprandial hyperglycemia.

1,5-AG is primarily excreted through urine, with 99.9% reabsorbed in the renal tubules by sodium/glucose cotransporter 4, encoded by the solute carrier family 5 member 9 gene [13,14]. When blood glucose levels exceed the renal threshold (>10 mmol/L), glucose competes with 1,5-AG for reabsorption, resulting in an increased urinary excretion of 1,5-AG and a decreased circulating concentration [15]. These mechanisms indicate that 1,5-AG can be a useful biomarker for detecting glucose intolerance, which is often challenging when using standard testing methods. While it is true that 1,5-AG levels are inversely related to blood glucose due to renal competition, this very mechanism underlies its clinical value in identifying postprandial hyperglycemia. Large-scale studies such as the Suita Study and others have validated its independent predictive power for cardiovascular events and diabetes risk, even in normoglycemic individuals. Rather than being used as a standalone marker, 1,5-AG may be more effective when used in conjunction with conventional indicators to maximize diagnostic sensitivity without compromising specificity.

Previous studies support these findings. A study with 359 individuals undergoing OGTT identified 102 cases of diabetes and determined that the optimal cutoff for 1,5-AG was 13.23 μg/mL (sensitivity: 89.7%, specificity: 73.5%). The sensitivity for detecting glucose intolerance was significantly higher when FBG was combined with 1,5-AG (47.1% vs. 97.1%) [16]. Another study of 2,184 participants found that the sensitivity for detecting glucose intolerance was significantly higher with the combination of HbA1c and 1,5-AG than with HbA1c alone (70% vs. 85%) [17]. These results are consistent with our findings, demonstrating that combining 1,5-AG with other indicators enhances the sensitivity in detecting glucose intolerance. In Japan, 1,5-AG testing is routinely used in clinical practice particularly in diabetes care. While 1,5-AG reflects glucose levels via competitive renal reabsorption, this characteristic enables it to sensitively capture transient postprandial hyperglycemia, which often escapes detection by FBG or HbA1c alone. The results of our study, which showed a significant increase in sensitivity when 1,5-AG was combined with either HbA1c or FBG (HbA1c + 1,5-AG: 72.2%, FBG + 1,5-AG: 72.2%), support its potential utility as a complementary biomarker in early-stage screening settings. This combined approach can reduce the likelihood of overlooking postprandial glucose excursions-key predictors of diabetes progression and cardiovascular events.

A study assessing the complexity and convenience of tests and involving 3,098 individuals (1,467 men and 1,631 women) found that 1,471 participants (47.5%) were diagnosed with diabetes. The accuracy of diabetes diagnosis improved when FBG with 1,5-AG was combined than with FBG alone, with an increase in the AUC from 0.781 to 0.912, sensitivity from 69.2% to 82.5%, and specificity from 72.3% to 83.5%. This combination also led to a 43.9% reduction in the need for an OGTT [18], indicating that using multiple tests tailored to different situations can help minimize patient burden.

In addition, it is important to track 1,5-AG levels over time. A 10-year follow-up study of individuals with IGT found a correlation between post-load blood glucose levels and 1,5-AG levels [19]. It has been suggested that a decrease in 1,5-AG levels over time could aid early detection of diabetes. Regular monitoring of 1,5-AG levels may contribute to a better diagnosis.

Managing postprandial blood glucose levels is vital for the prevention of diabetes. The study to prevent non-insulin-dependent diabetes mellitus trial studied the effect of the drug acarbose, an α-glucosidase inhibitor, in patients with IGT [20]. Out of the 1,368 participants, 37% developed diabetes. However, the acarbose group had a 32% incidence compared to 42% in the placebo group. Acarbose reduced the risk of type 2 diabetes by 25%. This suggests that controlling high blood glucose levels after meals may help delay diabetes in individuals with IGT.

CGM has gained popularity as an effective tool for identifying postprandial glucose spikes. In a study by Dehghani et al., CGM was used to examine 448 nondiabetic and 192 diabetic individuals. The results showed that 15% of healthy participants and 36% of the prediabetic group had blood glucose levels that reached the diabetic range [21]. Postprandial hyperglycemia is an independent risk factor for cardiovascular disease and has a stronger effect on health outcomes than fasting blood glucose levels [22]. The Diabetes Epidemiology: Collaborative analysis of diagnostic criteria in Europe study further confirmed that the 2-hour glucose level during an OGTT predicts the risk of cardiovascular diseases and overall mortality more accurately than fasting glucose levels [23]. In a 20-year follow-up study [24], participants were grouped into five categories based on their postprandial blood glucose levels. Those in the group with the highest levels two hours after eating had a 2.7 times higher risk of death than those in the group with the lowest levels. Postprandial hyperglycemia is linked to a higher risk of heart failure [25] and also increases the risk of atherosclerotic cardiovascular diseases [26,27]. Therefore, it is essential to monitor postprandial blood glucose levels. Although it would be ideal to monitor postprandial hyperglycemia in all individuals using CGM, there are limitations, such as its use in routine health check-ups. Studies have shown that 1,5-AG levels reflect blood glucose level fluctuations, similar to CGM [9]. Although it does not provide real-time blood glucose trends such as CGM, 1,5-AG could be a useful alternative for monitoring glucose changes in people without diabetes.

The type and objectives of health screening must also be considered when evaluating sensitivity and specificity [28]. Community health screenings, often organized by public healthcare systems, aim to lower overall mortality rates while minimizing the individual burden. This requires equal access to screening for all participants and emphasizes the need for high specificity to reduce potential harm. In contrast, voluntary screening, which is typically self-funded, prioritizes reducing individual disease risk and facilitating early detection, thus necessitating a greater focus on sensitivity to avoid missed cases. Our findings showed that combining 1,5-AG with voluntary screening improves sensitivity, indicating that using multiple indicators can reduce the chance of overlooking cases. Therefore, the incorporation of 1,5-AG is a valuable strategy.

This study has several limitations. First, the participants were recruited from among individuals seeking voluntary screening, which may have introduced a selection bias. In addition, the small sample size and the fact that the study was conducted in a single local city highlight the need for future studies involving larger samples from diverse regions to obtain more conclusive results. In addition, the lack of subgroup analysis due to the sample size constraint may limit the generalizability of the results. Future studies should explore the performance of 1,5-AG in stratified analyses, including sex-based or renal function-based subgroups, to further elucidate its diagnostic utility.

## Conclusions

The incorporation of 1,5-AG into a diagnostic testing panel may enhance the sensitivity of detecting postprandial blood glucose elevation in individuals with IGT. This suggests a potential reduction in the risk of overlooking patients who are at risk of postprandial hyperglycemia.

Given its ability to complement existing markers such as HbA1c and fasting blood glucose, 1,5-AG may serve as a practical tool in early screening strategies. Its use may be particularly valuable in settings where continuous glucose monitoring is not feasible. Further integration of 1,5-AG into routine health evaluations could support earlier identification and intervention in prediabetic individuals.
